# Association between serum adipocyte fatty-acid binding protein concentrations, left ventricular function and myocardial perfusion abnormalities in patients with coronary artery disease

**DOI:** 10.1186/1475-2840-12-105

**Published:** 2013-07-17

**Authors:** Chi-Lun Huang, Yen-Wen Wu, Chih-Cheng Wu, Lin Lin, Yu-Chin Wu, Pei-Ying Hsu, Yuh-Shiun Jong, Wei-Shiung Yang

**Affiliations:** 1 Department of Internal Medicine, Taoyuan General Hospital, Taoyuan, Taiwan; 2 Department of Internal Medicine, National Taiwan University Hospital, Taipei, Taiwan; 3 Department of Nuclear Medicine, National Taiwan University Hospital, Taipei, Taiwan; 4 Graduate Institute of Clinical Medicine, College of Medicine, National Taiwan University, No.7, Chung-Shan S. Rd., Taipei 100, Taiwan; 5 Department of Nuclear Medicine and Cardiology Division of Cardiovascular Medical Center, Far Eastern Memorial Hospital, No.21, Sec.2, Nanya S. Rd., Banciao Dist., New Taipei City, Taiwan; 6 National Yang-Ming University School of Medicine, Taipei, Taiwan; 7 Department of Internal Medicine, National Taiwan University Hospital Hsin-Chu branch, Hsinchu City, Taiwan; 8 Department of Nuclear Medicine, National Taiwan University Hospital Hsin-Chu branch, Hsinchu City, Taiwan; 9 Department of Nuclear Medicine, National Taiwan University Hospital Yun-Lin Branch, Douliou City, Taiwan

**Keywords:** Adipocyte fatty-acid binding protein, Coronary artery disease, Single-photon emission computed tomography

## Abstract

**Background:**

Adipokines, including adipocyte fatty acid-binding protein (A-FABP), have been demonstrated to be involved in the pathogenesis of atherosclerosis. In the present study, we investigated the association of circulating A-FABP level with severity of myocardial perfusion abnormalities analyzed by Tl-201 dipyridamole single-photon emission computed tomography.

**Methods:**

A total of 170 patients with coronary artery disease (CAD) from cardiovascular clinics were enrolled in the study. Serum A-FABP levels, echocardiography, and stress myocardial perfusion imaging results were analyzed.

**Results:**

Compared with the patients with mild CAD (summed stress score [SSS] ≤ 8), those with moderate to severe CAD (SSS > 8) had significantly higher A-FABP concentrations. However, the difference was attenuated in the subgroup of patients with heart failure. In the correlation analyses, A-FABP level was correlated with age, body mass index, waist circumference, levels of creatinine, fasting glucose, high-sensitivity C-reactive protein, N-terminal pro-brain natriuretic peptide, adiponectin, and several echocardiographic parameters, including left ventricular ejection fraction. Multivariate logistic regression analysis demonstrated that the A-FABP level was not only associated with higher SSS (odds ratio, 1.30; 95% confidence interval [CI], 1.01–1.69; *P* = 0.048), but also an independent risk factor for heart failure (odds ratio 2.71, 95% CI, 1.23–5.94; *P* = 0.013).

**Conclusions:**

Serum A-FABP levels not only were associated with myocardial perfusion abnormalities and left ventricular function, but also predicted the presence of heart failure in our patients with CAD.

## Background

Insulin resistance is closely associated with cardiovascular disease and heart failure (HF), and dysregulated adipokines, which are mainly released from adipose tissues, suggesting possible links between these conditions [1,2]. These adipokines, including adiponectin, resistin, and leptin, are known to mediate important inflammatory and metabolic responses [3-5]. Adipocyte fatty-acid binding protein (A-FABP), also termed aP2, is one of the most abundant intracellular lipid transport proteins in mature adipocytes and macrophages. However, data from rodents and humans suggest that it is also secreted into the bloodstream by adipose tissue [6]. In animal studies, A-FABP has been shown to regulate many inflammatory cytokines, mediate lipotoxicity and endoplasmic reticulum stress, and lead to endothelial dysfunction by impairing the nitric oxide pathway [7-9]. Recent human studies have confirmed its association with diabetes mellitus, non-alcoholic fatty liver disease, and cardiovascular disease. Circulating A-FABP level is also an independent predictor of metabolic syndrome development and coronary heart disease outcomes [10-14].

Two Asian studies showed a positive correlation between circulating A-FABP level and the severity of coronary artery disease (CAD), as determined by coronary angiography [15,16]. Tl-201 dipyridamole single-photon emission computed tomography (SPECT) is currently the standard tool in clinical CAD evaluation and demonstrates the extent of myocardial scar and ischemic burden. The aim of our study was to evaluate the correlation of circulating A-FABP levels with SPECT results, left ventricular function, and HF in CAD patients.

## Methods

### Study design

This cross-sectional study enrolled 170 consecutive patients with CAD from the cardiovascular outpatient clinics at Taoyuan General Hospital and National Taiwan University Hospital Hsin-Chu branch between July 2010 and June 2011. The study was approved by the institutional review board of both hospitals, and written informed consent was obtained from each patient before enrollment. Eligible patients had at least one of the following: old myocardial infarction (> 6 months), coronary revascularization, angiographic evidence of at least 50% stenosis in 1 or more major coronary arteries, or evidence of ischemia/scar on nuclear stress myocardial perfusion imaging. Patients were excluded if they had evidence of acute inflammatory or infectious disease, decompensated liver disease, end-stage renal disease, active malignancy, acute coronary syndrome, or a stroke within the month before the investigation.

All demographic information, including height, weight, waist measurements, cardiovascular risk factors, comorbid conditions, and a list of current medications were obtained from the patients’ medical records. Two-dimensional echocardiography and Tl-201 dipyridamole SPECT, as previously described [17], were performed in all participants. Left atrial dimension (LAd), left ventricular end-diastolic dimension (LVEDd), left ventricular end-systolic dimension (LVESd), interventricular septal dimension (IVSd), and left ventricular ejection fraction (LVEF) were evaluated and recorded in a blinded manner. Regional myocardial uptake was normalized and assessed using the 17-segment model and a semiquantitative scoring system of defect severity with a 5-point scoring system (0 = normal, 1 = equivocal, 2 = moderate, 3 = severe, and 4 = apparent absence of tracer uptake) and extent, as recommended by the American Society of Nuclear Cardiology [18]. Summed scores were calculated from the segmental scores, including a summed rest score (SRS; the sum of the 17 segmental rest scores) and summed stress score (SSS; the sum of the 17 segmental stress scores). A summed difference score (SDS; the difference between SSS and SRS) was also calculated. CAD severity, which was determined by SSS, was considered normal if the SSS < 4, mildly abnormal if the SSS was between 4 and 8, and moderately to severely abnormal if the SSS > 8. Image interpretation according to these definitions was performed by 2 experienced readers. Divergent interpretations were classified by consensus. The HF diagnosis was based on the criteria established in the American College of Cardiology/American Heart Association 2005 Guideline Update for the Diagnosis and Management of HF [19].

Laboratory examinations included renal function, fasting glucose, lipid profiles, high-sensitivity C-reactive protein (hsCRP), and N-terminal pro-brain natriuretic peptide (NT-proBNP). Glomerular filtration rate (GFR) was estimated using the formula from Modification of Diet in Renal Disease (MDRD). Serum A-FABP (BioVendor Laboratory Medicine, Inc. Brno, Czech Republic) and adiponectin concentrations (B-Bridge International, Inc., Cupertino, CA, USA) were analyzed by an enzyme-linked immunosorbent assay method, according to the manufacturer’s instructions.

### Statistical analysis

Data are reported as mean ± SD for normal distributions or as median with interquartile ranges for skewed variables. Data that were not normally distributed were logarithmically transformed before analysis. Group comparisons were performed by 2-sample *t* test. Univariate relationships between A-FABP level and clinical variables, serum biomarkers, and parameters derived from echocardiography and SPECT were assessed using Pearson’s correlation coefficient (r). Differences in serum A-FABP concentrations across SSS were compared by one-way analysis of variance, followed by the Bonferroni post-hoc test. To determine the independent predictors of myocardial perfusion abnormality and HF, the various parameters were included in multiple logistic regression analyses (ordered logistic regression for SSS). Analyses were performed using the STATA statistical software (release 10.0, StataCorp, College Station, TX, USA). All statistical tests were two-sided, where *P* < 0.05 was considered statistically significant.

## Results

The characteristics of all the participants are shown in Table 1. The mean age was 66.8 years, and 73% of the patients were male. The prevalence of hypertension and diabetes were 43% (n = 73) and 21% (n = 36), respectively. Forty-six patients (27%) had a history of myocardial infarction. Compared with those with mild CAD (SSS ≤ 8), the patients with moderate to severe CAD (SSS > 8) tended to be older, have a higher prevalence of diabetes, and have a lower LVEF. The serum creatinine, hsCRP, NT-proBNP, and A-FABP levels were also significantly higher in the patients with moderate to severe CAD. Among these patients, 34% (n = 58) had a clinical diagnosis of heart failure with New York Heart Association (NYHA) functional class II to IV. The patients with HF had significantly higher serum NT-proBNP and hsCRP levels than those without HF (*P* < 0.001 and *P* < 0.003, respectively). Adiponectin and A-FABP concentrations were also significantly higher in the patients with HF (HF vs. non-HF: median level, adiponectin, 12.1 vs. 6.5 mg/L, *P* < 0.0001; A-FABP, 34.8 vs. 21.0 ng/mL, *P* < 0.0001).

**Table 1:** Characteristics of coronary artery disease patients

**Parameter**	**All**	**Mild CAD**	**Moderate-severe CAD**	**P value**
	**(n =170)**	**(SSS ≤8, n = 88)**	**(SSS >8, n = 82)**	
Age (yr)	65.7 ± 13.6	64.0 ± 13.1	67.6 ± 13.9	0.09
Male gender (%)	115 (68%)	55 (63%)	50 (73%)	0.14
Body mass index (kg/m^2^)	24.9 ± 3.7	24.9 ± 3.5	24.9 ± 4.1	0.99
Waist (cm)	85 ± 11	86 ± 12	84 ± 11	0.41
Hypertension (%)	73 (43%)	36 (41%)	37 (45%)	0.58
Diabetes mellitus (%)	36 (21%)	14 (16%)	22 (27%)	0.08
Smoking (%)	43 (25%)	14 (16%)	29 (35%)	0.004
LVEF	0.61 ± 0.16	0.68 ± 0.13	0.53 ± 0.16	< 0.0001
Creatinine (mg/dL)	1.13 ± 0.45	1.03 ± 0.34	1.22 ± 0.53	0.005
Fasting glucose (mg/dL)	116 ± 45	110 ± 42	123 ± 47	0.07
Total cholesterol (mg/dL)	186 ± 47	184 ± 38	189 ± 57	0.58
HDL-C (mg/dL)	48 ± 16	51 ± 18	44 ± 12	0.006
LDL-C (mg/dL)	108 ± 34	105 ± 26	111 ± 41	0.25
ALT (U/L)	29 ± 19	27 ± 19	30 ± 20	0.18
hsCRP (ug/mL)*	2.3 (1.1, 8.2)	1.8 (0.6, 4.6)	3.0 (1.7, 11.9)	0.0005
NT-proBNP (ng/L)*	107 (42, 797)	63 (38, 127)	797 (129, 2572)	< 0.0001
Adiponectin (mg/L)*	7.6 (5.2, 12.3)	7.4 (5.0, 10.0)	7.7 (5.3, 15.7)	0.13
A-FABP (ng/mL)*	26.2 (17.2, 40.1)	21.1 (16.2, 33.8)	32.4 (19.7, 48.5)	0.002
Medications				
ACEi/ARBs	101 (59%)	34 (39%)	67 (82%)	< 0.0001
Beta-blockers	57 (34%)	23 (26%)	34 (41%)	0.03
CCBs	39 (23%)	24 (27%)	15 (18%)	0.16
Diuretics	51 (30%)	14 (16%)	37 (45%)	< 0.0001
Statins	36 (21%)	12 (14%)	24 (29%)	0.01
Metformin	12 (7%)	5 (6%)	7 (9%)	0.47

Values are expressed as mean ± S.D., median (25^th^ - 75^th^ percentile), or n (percentage).

*Logarithmically transformed before analysis.

ACEi/ARBs, angiotensin-converting enzyme inhibitor / angiotensin II receptor blockers; A-FABP, adipocyte fatty acid-binding protein, ALT, alanine aminotransferase; CCBs, calcium channel blockers; HDL-C, high-density lipoprotein cholesterol; hsCRP, high-sensitivity C-reactive protein; LDL-C, low-density lipoprotein cholesterol; LVEF, left ventricular ejection fraction.

Table 2 shows the results of the correlation analysis between the sex-adjusted A-FABP level and other clinical parameters. In the overall patient population, after the adjustment for sex, the A-FABP level was positively correlated with age, body mass index (BMI), waist circumference, levels of serum creatinine, fasting glucose, high-density lipoprotein cholesterol (HDL-C), hsCRP, adiponectin, and NT-proBNP, but negatively correlated with MDRD-GFR. Among the parameters derived from echocardiography, A-FABP level was inversely correlated with LVEF (r = −0.33, *P* < 0.0001), but positively correlated with LVEDd, LVESd, LAd, and IVSd.

**Table 2:** Correlation of sex-adjusted A-FABP levels with biochemical parameters, echocardiographic indexes and myocardial perfusion abnormalities

	**r**	**P**
Age	0.33	< 0.0001
Body mass index	0.31	0.0001
Waist circumference	0.36	< 0.0001
GFR	−0.48	< 0.0001
Fasting glucose	0.18	0.024
Total cholesterol	0.10	0.186
HDL-C	−0.20	0.013
LDL-C	0.02	0.847
hsCRP*	0.32	0.0001
NT-proBNP*	0.49	< 0.0001
Adiponectin*	0.20	0.019
**Echocardiography**		
LVEF	−0.33	< 0.0001
LVEDd	0.29	0.0002
LVESd	0.35	< 0.0001
LAd	0.33	0.0003
IVSd	0.40	< 0.0001
**SPECT**		
Summed rest score (SRS)	0.13	0.088
Summed stress score (SSS)	0.19	0.013
Summed difference score (SDS)	0.17	0.027

* Logarithmically transformed before analysis.

GFR, glomerular filtration rate; HDL-C, high-density lipoprotein cholesterol; IVSd, interventricular septal dimension; LAd, left atrial dimension; LVEDd, left ventricular end-diastolic dimension; LVESd, left ventricular end-systolic dimension; LVEF, left ventricular ejection fraction; LDL-C: low-density lipoprotein cholesterol.

**Figure 1:**
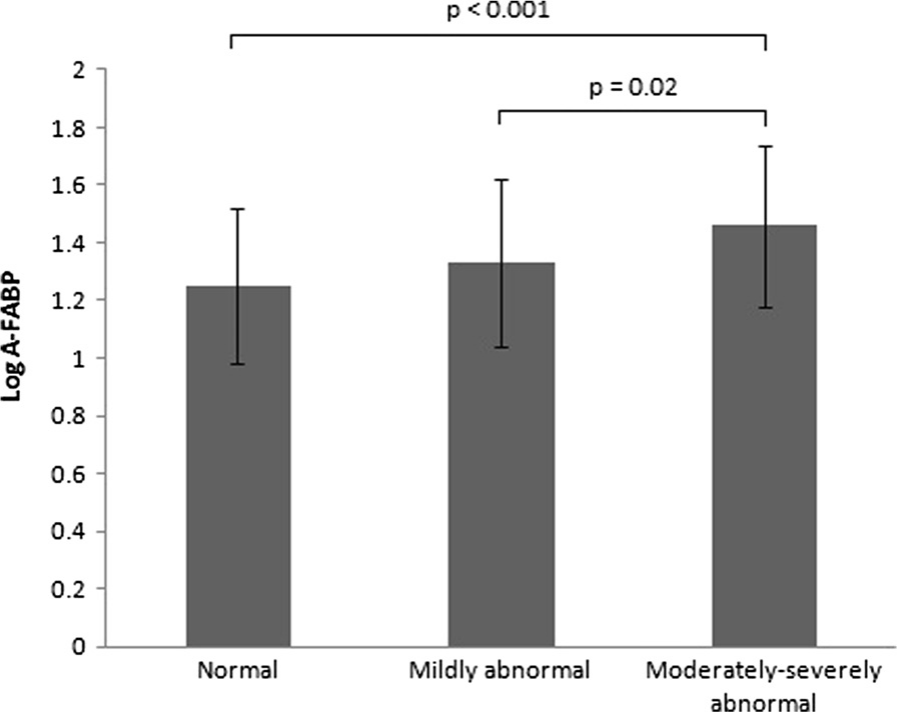
**Association between serum A-FABP concentration and coronary artery disease severity determined by summed stress score (SSS).** Normal: SSS < 4, mildly abnormal: 4 ≤ SSS ≤ 8, moderately to severely abnormal: SSS > 8.

In the analysis of SPECT results, SRS, SSS, and SDS were regarded as indicators of myocardial scar, CAD severity, and ischemic burden, among which SSS and SDS showed a positive correlation with A-FABP levels (r = 0.19 and 0.17, respectively; Table 2). The patients with a high scar burden (SRS ≥ 4) had significantly higher A-FABP levels (median, 31.6 vs. 21 ng/mL, *P* = 0.006) than those with low or no scar burden (SRS < 4). Compared with those with normal SSS (SSS < 4), a trend of higher A-FABP levels was observed in the patients with more extensive CAD (SSS ≥ 4, *P* = 0.008; Figure 1). Multivariate ordered logistic regression, using SSS (normal, mildly abnormal, and moderately to severely abnormal) as dependent variable, was performed to investigate the association of A-FABP level and myocardial perfusion abnormality. After adjustment for age, sex, hypertension, BMI, smoking status, level of fasting glucose, creatinine, low-density lipoprotein cholesterol (LDL-C), and hsCRP, A-FABP level was still an independent predictor of higher SSS (odds ratio, 1.30 per 10-ng/mL increase; 95% confidence interval [CI], 1.01–1.69; *P* = 0.048). However, we further analyzed the association between A-FABP level and myocardial perfusion abnormalities in the subgroups of the patients with and without HF. The differences in A-FABP levels between patients with high and low SSS, and also SRS, were significantly attenuated in those with concomitant HF (Table 3).

**Table 3:** Serum A-FABP distribution according to summed stress score, summed rest score and heart failure (HF) condition

	**A-FABP (ng/mL)**					
	**All (n = 170)**		**Non-HF (n = 112)**		**HF (n = 58)**	
Summed stress score						
SSS ≤ 8	21.1 (16.2, 33.8)	P = 0.002	20.6 (15.5, 31.6)	P = 0.037	35.1 (29.1, 58.3)	P = 0.801
SSS > 8	32.4 (19.7, 48.5)		27.6 (16.2, 41.0)		34.5 (23.2, 53)	
Summed rest score						
SRS < 4	21 (15.3, 33.9)	P = 0.006	20.6 (14.5, 32.0)	P = 0.039	33.9 (28.0, 74.3)	P = 0.760
SRS ≥ 4	31.6 (19.2, 45.7)		25.7 (17.1, 40.7)		35.0 (22.4, 53.6)	

Presented with median (25^th^ - 75^th^ percentile) and logarithmically transformed before analysis.

Finally, multiple logistic regression analysis was performed to accurately evaluate the relationship between A-FABP level and HF in the subjects with CAD. A-FABP level was also independently associated with the presence of HF, with an odds ratio of 2.71 (95% CI, 1.23–5.94; *P* = 0.013) for each 10-ng/mL increase (Table 4).

**Table 4:** Multiple logistic regression analysis showing odds ratio for the risk of heart failure in patients with coronary artery disease

**Model**	**A-FABP (per 10 ng/mL increase)**	**OR**	**95% CI**	**P value**
Model 1	Adjusting for age and sex	1.58	1.25 – 1.99	< 0.001
Model 2	Adjusting for age, sex, BMI, smoking, hypertension, fasting glucose, LDL-C, creatinine and hsCRP	2.03	1.31 – 3.14	0.002
Model 3	Adjusting for age, sex, BMI, smoking, hypertension, fasting glucose, LDL-C, creatinine, hsCRP, adiponectin, SSS, SRS, ACEi/ARBs, diuretics and statins use	2.71	1.23 – 5.94	0.013

ACEi/ARBs, angiotensin-converting enzyme inhibitor / angiotensin II receptor blockers; BMI, body mass index; hsCRP, high-sensitivity C-reactive protein; LDL-C, low-density lipoprotein cholesterol; SRS, summed rest score; SSS, summed stress score.

## Discussion

Our study demonstrated that serum A-FABP levels were associated with CAD severity, as determined by SPECT. We also found that the patients with HF had higher A-FABP levels than those without HF. After multivariate adjustment, A-FABP was an independent predictor of myocardial perfusion abnormalities and HF. However, the correlations between A-FABP level and myocardial perfusion abnormalities were attenuated in the subgroup of patients with HF.

A-FABP expression is known to be a key proinflammatory mediator that links obesity with cardiovascular disease. Data from animal to human studies all support its pathological roles in atherosclerosis. In apoE knockout mice, A-FABP deficiency resulted in a marked reduction of aortic atherosclerotic lesions [20]. In addition, pharmacological inhibition of A-FABP also rendered significant protection against atherosclerotic plaque formation [21]. Depletion of A-FABP expression prevented oxidized LDL-induced foam cell formation by increasing cholesterol efflux and also inhibited IkB kinase/NF-kB activity. This finding further supported the proatherogenic effects of A-FABP in macrophages. In addition, A-FABP inhibited eNOS activation and nitric oxide production in vascular endothelial cells, which led to endothelial dysfunction. This suppressive effect was reversed by treatment with an A-FABP inhibitor [9,22]. In human studies, A-FABP is known to be involved in metabolic syndrome and cardiovascular diseases. Circulating A-FABP levels have been shown to correlate with the presence of CAD and carotid atherosclerosis, with the number of diseased vessels, and with plaque burden of coronary arteries [15,16,23,24]. It is also an important predictor of cardiovascular outcomes in patients with coronary heart disease, acute ischemic stroke, and end-stage renal disease [14,23,25]. Locally expressed A-FABP in atherosclerotic plaques also reduces plaque stability [26].

We also found an association between circulating A-FABP level and metabolic components. A-FABP level showed a positive correlation with waist circumference and fasting glucose level but a negative correlation with HDL-C. A weak correlation was also observed between concentrations of A-FABP and adiponectin, another well-documented insulin resistance marker. The A-FABP concentration also correlated positively with the inflammatory marker hsCRP, as previous studies have demonstrated. There were significant differences in A-FABP levels between men and women. Two Asian studies made different conclusions about the relationship of A-FABP and CAD in different sexes. In our study, the differences in A-FABP levels between patients with mild and severe CAD were consistent, although the differences were more significant in women (median, 44.6 vs. 26.3 ng/mL, *P* = 0.0006). Previous studies have also shown that atorvastatin and olmesartan treatment lowered circulating A-FABP levels [27,28]. The higher A-FABP levels in our patients treated with statins and angiotensin-converting enzyme inhibitors or angiotensin II receptor blockers might be related to the higher prevalence of metabolic syndrome, severe CAD, and advanced HF. It is interesting that both HF and non-HF subgroup patients taking thiazide diuretics had significantly higher A-FABP concentrations. Thus, the effects of thiazide diuretics, which are known to cause metabolic abnormalities, on serum A-FABP concentrations would be worth investigating in a prospective, interventional study design.

The upregulation of A-FABP expression and other adipokines in HF has also been demonstrated in recent studies [3,4,29,30]. However, their exact roles in the pathogenesis of HF remain unclear. The complex neurohormonal and metabolic abnormalities associated with HF have received increased attention. Of note, upregulation of inflammatory cytokines, catecholamines, growth hormone, and catabolic steroids is known to mediate increased lipolysis and insulin resistance [31]. Data from randomized controlled trials of HF have suggested a diabetes prevalence of 8% to 41%, and insulin resistance is found to correlate with the functional, clinical, and biochemical severity of HF [32]. Most importantly, a recent in vitro study by Lamounier-Zepter et al. demonstrated that A-FABP suppressed the contraction of cardiomyocytes by attenuating intracellular Ca^2+^ levels [33]. This offered strong evidence that A-FABP might be directly involved in the pathogenesis of HF. In human studies, Mingya et al. [29] demonstrated that serum A-FABP levels were associated with HF severity, as determined by the NYHA classification system, and were positively correlated with NT-proBNP levels in Chinese subjects. A further large-scale prospective study showed that plasma concentration of A-FABP predicted a 1.09-fold higher risk of heart failure during a median follow-up of 10.7 years [30]. In our patients, the moderate correlation between A-FABP and NT-proBNP levels also suggested a link between A-FABP level and HF severity. Because the association between A-FABP level and CAD severity was attenuated in the subgroup of patients with HF and the predictive value of A-FABP to HF persisted after adjustment for SRS and SSS, we believe that HF-related metabolic disarrangement, rather than CAD severity, may be a more important determinant of A-FABP levels in CAD patients with HF.

Notably, higher serum creatinine levels are observed in moderate to severe CAD, with renal function being the main independent predictor of circulating A-FABP levels. Previous studies have shown a negative relationship between GFR and A-FABP concentration, suggesting that A-FABP might have an important role in the interplay between renal dysfunction and the development of coronary atherosclerosis [34]. Because A-FABP is a low-molecular-weight plasma protein, freely filtered at the glomerulus, a decrease in glomerular function will result in an elevation of A-FABP concentration. In our current study, A-FABP level remained an independent predictor of myocardial perfusion abnormality, even after adjusting for renal function.

There were some limitations in the present study. First, the small number of patients enrolled was inadequate to obtain conclusive data. Second, owing to the cross-sectional design of our study, some conclusions were based on findings from association studies that did not imply a direct causal relationship. Third, the SSS could not accurately indicate the severity of CAD in a small number of patients who underwent percutaneous coronary angioplasty before SPECT analysis. However, the myocardial scar, indicated by SRS, was not affected by whether angioplasty had been performed.

## Conclusions

In our study, the serum A-FABP levels not only were associated with myocardial perfusion abnormalities and left ventricular function, but also predicted the presence of HF in the CAD patients. In conjunction with previous data, our results confirm the important role of A-FABP in metabolic syndrome, atherosclerosis, and HF. However, a further large-scale, prospective study is needed to confirm its predictive value upon traditional CAD risk factors.

## Abbreviations

A-FABP: Adipocyte fatty acid-binding protein; BMI: Body-mass index; CAD: Coronary artery disease; HF: Heart failure; GFR: Glomerular filtration rate; HDL-C: High-density lipoprotein cholesterol; hsCRP: High-sensitivity C-reactive protein; IVSd: Interventricular septal dimension; LAd: Left atrial dimension; LVEDd: Left ventricular end-diastolic dimension; LVESd: Left ventricular end-systolic dimension; LVEF: Left ventricular ejection fraction; LDL-C: Low-density lipoprotein cholesterol; NT-proBNP: N-terminal pro-brain natriuretic peptide; NYHA: New York Heart Association; SPECT: Single-photon emission computed tomography; SDS: Summed difference score; SRS: Summed rest score; SSS: Summed stress score.

## Competing interests

The authors declared no competing interests.

## Authors' contributions

CLH, YWW, CCW, LL and YSJ conceived the study, participated in study design and coordination. YCW and PYH analyzed the SPECT. CLH, YWW and WSY assisted with the preparation and critical review of this manuscript. All authors read and approved the submitted manuscript.
